# Characterization of the Coding Sequence of the *MC1R* (Melanocortin 1 Receptor) Gene of Ayam Cemani Black Chickens

**DOI:** 10.3390/ani14172507

**Published:** 2024-08-29

**Authors:** Beata Horecka, Witold Wojciechowski, Kamil Drabik, Karolina Wengerska, Justyna Batkowska

**Affiliations:** Institute of Biological Basis of Animal Production, University of Life Sciences in Lublin, 20-950 Lublin, Polandjustyna.batkowska@up.lublin.pl (J.B.)

**Keywords:** plumage color, missense substitution, haplotype, black chickens, breed identification

## Abstract

**Simple Summary:**

The genetic background of color is a very complex issue in both birds and mammals. Color-related genes may be represented by multiple alleles and show many interactions. Black chicken breeds are special due to their solid uniform pigmentation, which in the case of Ayam Cemani also concerns the bones and skin. One of the crucial genes involved in the melanogenesis pathway is *MC1R* (melanocortin 1 receptor). It is a one-exon gene that has been already studied in many different species and breeds. In the case of chicken breeds, there are numerous alleles of *MC1R* and they have a significant impact on the final plumage color of the individual. Here, the Ayam Cemani *MC1R* complete coding sequence is characterized for the first time. A new *MC1R* missense substitution has enabled us to distinguish a new haplotype/extended black allele. This is important in terms of completing and supporting current knowledge concerning the genetic background of plumage control, as well as in relation to describing genetic diversity between different breeds.

**Abstract:**

Plumage color is one of the most important traits characterizing chicken breeds. Black-boned chickens constitute a specific group of breeds with a unique phenotype. One of the representatives is the Indonesian Ayam Cemani. The extraordinary black phenotype results from a specific chromosomal rearrangement. We used complete CDS of crucial color-related gene *MC1R*, which plays a key role in melanin distribution but has not been previously studied in Ayam Cemani. It turned out that Ayam Cemani individuals possess a newly found non-synonymous mutation G355A resulting in amino acid substitution D119N. Together with the presence of G274A (E92K), the new missense variant enabled us to distinguish a new extended black allele at the E locus. All of the investigated birds were heterozygous in terms of the new mutation. Previous studies and our own results indicate a high level of genetic variation within the *MC1R* gene within and between chicken breeds. Besides the key mutations that make it possible to distinguish particular major alleles, there are also numerous substitutions that give haplotypes more characteristics for individual breeds.

## 1. Introduction

In chickens, the process of domestication from a single ancestor, the Red Jungle Fowl (*Gallus gallus*), has created a wide variety of plumage colors. The feather color of poultry is an important feature of different breeds, and has always been valued by breeders and poultry producers.

The black-boned chicken (BBC) breeds occur globally and have distinct names, such as Ayam Cemani (Indonesia), Black H’Mong (Vietnam), Tuzo (Argentina), Svarthöna (Sweden) [1,2], Yeonsan Ogye (Korea) [3], and Thai BBC (Thailand) [4]. China has a high diversity of BBC breeds, including Silkie, Jiangshan, Lueyang, Sichuan, Xingwen, Yugan, Dehua, Jinhu, Muchuan, Wumeng, Yanjin, Xichuan, Tuanfu, Wuliangshan, Emei, and Miyi fowl [5,6,7,8,9]. India has a single breed of BBC, commonly known as Kadaknath [10].

The black-boned chicken phenotype results from melanin deposition throughout the body—melanin hyperpigmentation or fibromelanosis caused by the Fm allele [11]. Modern studies indicate that a chromosomal rearrangement on chromosome 20 is involved in the Fm locus [2,3,12]. The overexpression of the endothelin-3 (*EDN3*) gene located within the Fm locus is responsible for hyperpigmentation present in the black-boned chicken [12,13,14].

The Indonesian Ayam Cemani also exhibits fibromelanosis (Fm) or dermal hyperpigmentation and possesses complex segmental duplications on chromosome 20 that involve the endothelin 3 gene, *EDN3*. A genomic region, *DR1* of 127 kb, together with another region, *DR2* of 171 kb, was duplicated by unequal crossing over, accompanied by inversion of one *DR2*. Quantitative PCR and copy number variation analyses on the Ayam Cemani genome sequence confirmed the duplication of *EDN3*. These genetic arrangements are identical in Ayam Cemani and Silkie, indicating a single origin of the genetic cause of Fm [2]. Recent research [15] has revealed that Ayam Cemani individuals might be both homozygous (Fm/Fm) and heterozygous (Fm/fm+). The results also showed that mostly, the phenotype for Ayam Cemani with (Fm/fm+) genotype is reddish black in their comb; meanwhile, the individuals with (Fm/Fm) genotype showed heavy black pigmentation. The data from whole-genome resequencing have established that Chinese and Korean BBC breeds with Kadaknath native to India all share the complex chromosomal rearrangement junctions at the fibromelanosis (Fm) locus [16].

Ayam Cemani chickens are very unique as they are entirely black, both externally and internally. The effect of fibromelanosis is visible not only in the plumage but it also concerns comb, shank, tongue, and eye. What is more, it is possible to find this hyperpigmentation in the innermost parts of the body: in the muscles, intestines, bones, peritoneum, and also trachea. The cause of this phenomenon is the fact that usually, in most chicken breeds, melanocytes are the only cells able to release pigmentation. In the Ayam Cemani chicken, all kind of cells, not only melanocytes, are able to release pigment and there is an accumulation of melanin in the internal organs and connective tissue [17].

There are several basic protein-coding genes that control the plumage color of chickens. MC1R (melanocortin 1 receptor) is a G protein-coupled receptor located on the plasma membrane of melanocytes and has seven transmembrane domains. In chickens, the E locus encodes the *MC1R* protein consisting of 314 amino acids. The gene is located on chromosome 11 and has a 945 bp long CDS region which shares 64% identity with its counterpart in mammals [18,19]. The gene affects the distribution of the two melanin pigments (eumelanin and phaeomelanin) in feathers, and is important for accurate down color sexing.

**Figure 1:**
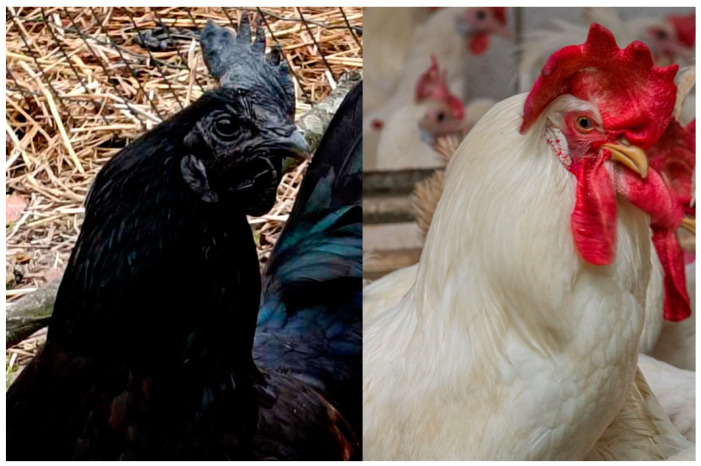
Example of extremely different color phenotypes of Ayam Cemani and White Leghorn chickens with different sequence variants of *MC1R* extended black allele.

*MC1R* (melanocortin 1 receptor) is one of the most important color-related genes, although it is not entirely clear which mutations are associated with each phenotype. It seems that mutation Glu92Lys leads an active receptor to produce eumelanin. It was observed [20] that this same mutation may be necessary to determine the extended black and the buttercup phenotypes in chickens, in addition to another different mutation in each one. A recent comprehensive study [18] based on previously published data, and the authors’ own research, revealed the presence of 14 haplotypes of *MC1R* gene in native and non-Japanese chicken breeds. Six of them were extended black haplotypes discovered in a variety of differently feathered chicken breeds (Figure 1) [18,20,21,22,23]. In turn, a previous study [24] characterized *MC1R* alleles in Chinese black chickens and described the dominant Extended Chinese Black haplotype (E^C^).

Feather color is a genetic marker that can be used for determining hybrid combinations, the variety and purity of genetic relationships, and evaluating product quality. The study and knowledge of genes involved in plumage color have increased considerably, because these genes could provide genetic markers useful for the identification of breeds. It might be also a useful tool for determination of phylogenetic relationships and the breeding history. Ayam Cemani is an Indonesian black-boned breed sharing Fm rearrangement with Chinese, Korean, and Indian BBC representatives. It thus might be interesting to further investigate the phylogenetic relationships between them, applying additional color-related genetic markers.

Taking these issues into account, the aim of the study was to determine the sequence and the polymorphisms in the complete coding region of *MC1R* gene, covering previously described breed-specific mutations, in black chickens of the Ayam Cemani breed.

## 2. Materials and Methods

The biological material for the study were feathers (quills) from hens with black plumage of the Ayam Cemani breed—the tested group, kept by a private local breeder. Ten individuals were included in the research; two feathers were collected from each individual. These were non-invasive samples as the feathers left by the individuals were collected from the cages. DNA extraction was performed using the Genomic Mini kit by A&A Biotechnology according to manufacturer’s protocol.

The complete coding region was amplified using verified primers [20]. Two PCR products were obtained for each individual (one for each feather sample) to enable verification of identified mutations; in total, 20 specific amplicons were prepared for sequencing. The expected length of the PCR product was approximately 945 bp. Primer sequences were: MC1R_F (5′-ATCCTTGTGCCTGGGGTG-3′) and MC1R_R (5′-CATCCATCCATCCTCCTGTC-3′).

PCR was performed using a PCR Mix Plus kit (A&A Biotechnology, Gdańsk, Poland). The reaction was performed in a volume of 25 µL. A single sample contained 1 × PCR Mix Plus including: 4.0 mM MgCl_2_, 0.1 U/µL of Taq DNA Polymerase, and 0.5 mM of each dNTP, complemented by 0.2 µM of each primer and 3 µL of genomic DNA. PCR was performed on a Labcycler (SensoQuest, Göttingen, Germany) using the following thermal profile: 3 min at 95 °C prior to 35 cycles of 30 s at 95 °C, 60 s at 56 °C, and 60 s at 72 °C, followed by an extension step of 3 min at 72 °C. Spectrophotometric evaluation of the PCR product was performed on the NanoDrop One (Thermo Scientific, Waltham, MA USA). Electrophoresis of PCR products was performed on 2% agarose gel with TBE buffer and ethidium bromide, using the Gene Ruler 100 bp DNA Ladder (Fermentas, Waltham, MA, USA) at a constant voltage of 70 V for 180 min.

Sequencing of all obtained PCR products was performed by commercial company Genomed S.A. (Warsaw, Poland) using a special ‘Extra long run’ service with a guarantee of higher quality and length of readings, allowing us to obtain a result in the range of 1000–1100 nucleotides. In addition, F and R primers were used to sequence both strands and ensure better quality of reads.

Preliminary analysis of the sequencing results was carried out with DNA Baser (DNA Baser Sequence Assembler v. 3) and MEGA 11 [25] software. Sequence data were deposited in GenBank (accession number: PP959149 and PP959150). A phylogenetic tree was constructed in MEGA 11 using the maximum likelihood (ML) method. The phylogenetic distances were estimated with Hasegawa–Kishino–Yano (HKY) determined by the ‘Models’ option in the MEGA 11 program. The robustness of tree branches was determined by bootstrap analysis using 1000 re-samplings.

## 3. Results

The optimization of the PCR reaction resulted in an expected specific amplification product of approximately 1100 bp length from Ayam Cemani breed samples. The amplified fragment, processed by ends trimming after sequencing, covered the complete *MC1R* coding sequence of 945 bp.

**Figure 2:**
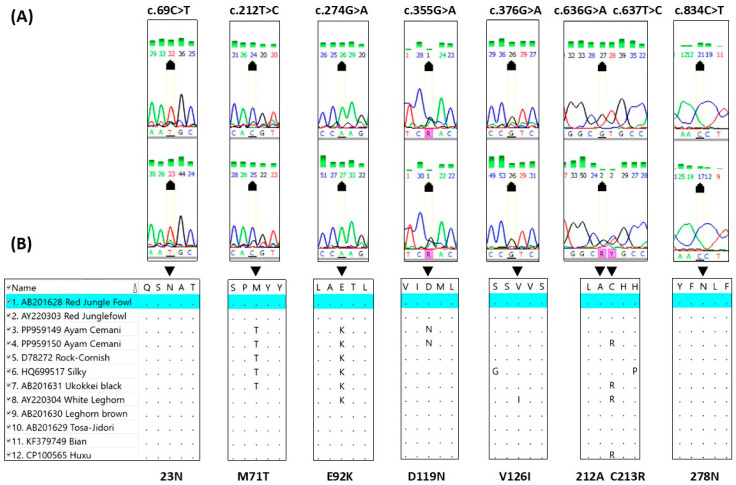
Sequence electropherograms for the seven key mutations in two described different variants of the Ayam Cemani *MC1R* sequence (**A**) and alignment of the MC1R protein regions including the deduced amino acid substitutions including the corresponding position of the mutated nucleotides with the same protein regions of other chicken breeds (**B**).

By comparing the investigated *MC1R* coding sequence of the Ayam Cemani chickens with the reference coding sequence of the Red Jungle Fowl, it was found that all the tested individuals were homozygous for the synonymous mutation C69T (genotype T/T). In Ayam Cemani chickens we also found missense mutation T212C that was observed in all ten investigated samples. The G274A substitution resulting in the E92K amino acid sequence change is obligatory for the black plumage expression. All investigated Ayam Cemani individuals were homozygous for the A allele at the position 274 of the coding sequence. All Ayam Cemani individuals were also homozygous for the G/G genotype at the position G376A. An interesting situation was observed in CDS positions 636 and 637. Six out of ten birds were characterized by homozygous GT combination in these positions. However, four other individuals had heterozygous set G/A at position 636 and T/C at 637. There was also one missense mutation in Ayam Cemani *MC1R* CDS that was not previously described. It was the G355A substitution, changing the amino acid from aspartic acid to asparagine (D119N). This missense mutation was found in all ten investigated animals; they were all G/A heterozygotes (Figure 2). It can be assumed that Ayam Cemani represents *MC1R* variants based on previously described mutations characteristic for extended black alleles and modified variants including the newly described G355A (D119N) missense mutation (Table 1).

**Figure 3:**
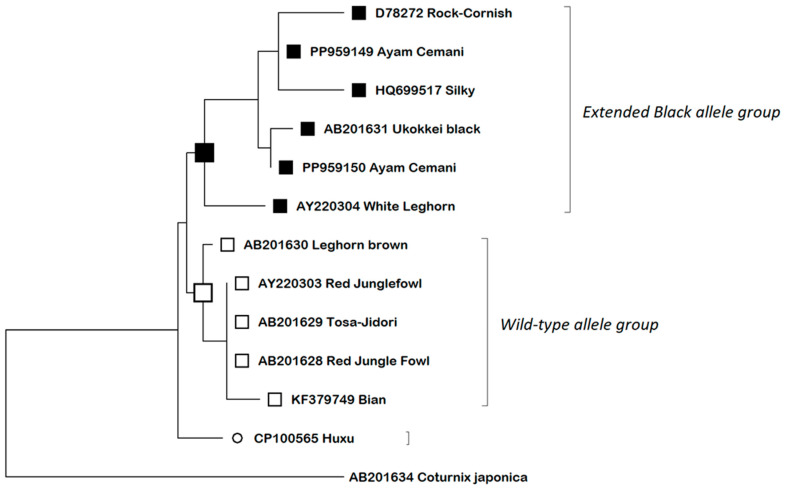
The phylogenetic tree constructed by ML method: sequences are described by breed name and GenBank accession number.

We used our own sequences of Ayam Cemani (PP959149 and PP959150) together with GenBank reference sequences of chickens representing variety of colors, with breed information specified in record metadata, to construct the ML phylogenetic tree (Figure 3). The Japanese quail (*Coturnix japonica*) *MC1R* sequence AB201634 was used as the outgroup. The aligned sequences collapsed into two main groups: ‘Wild-type allele group’ including Red Jungle Fowl, Tosa Jidori, Bian and Leghorn Brown; and ‘Extended Black allele group’ which consisted of the black chicken breeds Ukokkei Black and Ayam Cemani together with White Leghorn, Silky, and Rock-Cornish. Phylogenetic analyses performed in our own study were limited due to accessibility of GenBank reference sequences with reliable metadata concerning the breed. It showed that both Ayam Cemani MC1R sequence variants were significantly different. The PP959150 sequence was grouped together with the Ukokkei Black sequence while the PP959149 variant was more related to Rock-Cornish and Silky. It also showed its relatedness to extended black alleles present in White Leghorn, although they were clearly separated at the phylogenetic tree. The aligned Huxu sequence represented a separate branch at the phylogenetic tree, distinct in relation to both wild-type and extended black variants.

## 4. Discussion

Melanocortin 1 receptor (*MC1R*) is the major gene that controls chicken plumage color, including extended black (E), birchen (E^R^), dominant wheaten (e^wh^), recessive wheaten (e^y^), brown (e^b^), buttercup (e^bc^), and wt (e^+^) alleles. Specific mutations in the coding sequence of the *MC1R* gene make it possible to distinguish particular alleles.

It was shown that the extended black allele at the E locus is due to a missense mutation, G274A, leading to an E92K substitution in the MC1R peptide [20]. Most of extended black alleles also share the T212C missense mutation resulting in M71T substitution. Missense mutation H215P (A644C) may interfere with the constitutive activation of the receptor caused by E92K (and possibly M71T) blocking the expression of black plumage. A previous study [18] has confirmed that the A427G and G274A substitutions contribute in expressing brownish and black plumage color, respectively. It was also confirmed that the buttercup allele does not express black plumage despite possessing a G274A substitution, under the suppression effect of A644C. In contrast, the birds homozygous for the birchen allele, characterized by the lack of the G274A substitution, presented solid black plumage. The same study also presented a comprehensive data set regarding 14 known *MC1R* haplotypes. Three of them were newly found haplotypes, while two compromised the newly discovered C919G substitution. The most numerous groups included extended black haplotypes, as there have been six described so far [18,20,21,22,23].

The first Ayam Cemani *MC1R* sequence variant that was present in the majority (6 out of 10) of the investigated individuals, is based on a mutations set characteristic for the previously described H7 [21], with the additional G355A substitution. The four remaining birds possessed substitution variants also found previously in H8 [22] and H9 [18], but of course also supported by the G355A missense variant.

The *MC1R* coding sequence analyzed in our own research in Ayam Cemani samples was characterized by the nucleotide sets specific for previously described extended black alleles. However, within this fragment we found the new mutation G355A, resulting in an amino acid substitution and providing a new extended black haplotype exclusive for Ayam Cemani chickens. A dominant Extended Chinese black E^C^ (CAA) haplotype of three non-synonymous alleles has been described: T212C (M71T, rs312264213), G274A (E92K, rs314881228), and A644C (H215P, rs735789743), that is different from the E (TAA) haplotype in non-Chinese breeds. According to the findings [24], the E^C^ haplotype is dominant over the e^bc^ (CAC) and e^+^ (TGA) alleles. The Ayam Cemani haplotypes described in our own study are also composed of T212C, G274A, and A644C missense mutations. It suggests that Ayam Cemani haplotypes seem to be closely related to Extended Chinese Black E^C^ haplotype present in Chinese black chicken breeds.

The most frequent haplotype (frequency 0.48) in Ukokkei Black was H8, composed of T212C, G274A, G636A, and T637C missense mutations, and it has A at the position 644 which is characteristic also for Ayam Cemani [18]. In means that the Ukokkei Black *MC1R* sequence contains all non-synonymous substitutions [24] as characteristic for the Extended Chinese black E^C^ allele. However, considering the fact that the Indonesian Ayam Cemani also possesses the key mutations T212C, G274A, and A644C, and so the haplotype CAA, the E^C^ haplotype may turn out not to be an unambiguous genetic marker for Chinese black chicken breeds.

## 5. Conclusions

Based on our own findings supported by previously published results, it can be assumed that chicken breeds can be at least preliminarily identified using CDS of the *MC1R* gene. If the investigated samples are non-invasive or degraded and it is impossible to amplify the whole coding sequence, the crucial coding part includes the T212C and G274A missense mutations. It makes it possible to preliminarily distinguish between breeds possessing the extended black alleles variant and wild-type-like variants. However, the *MC1R* gene shows high levels of genetic variation; furthermore, numerous chicken breeds were not studied in terms of the gene sequence. Despite some key mutations, there are also numerous breed-specific substitutions, and Ayam Cemani G355A (D119N) can serve as the example. This indicates the need for further analysis of color-related genes in chickens.

## Figures and Tables

**Table 1:** Characteristics of sequence variants in the analyzed *MC1R* coding sequence of Ayam Cemani compared with previously described extended black allele haplotypes H7–H12.

***MC1R*** **Haplotype**	**Nucleotide Position**								**Reference**
	c.69	c.212	c.274	c.355	c.376	c.636	c.637	c.834	
	**Substitution Type**								
	C>T	T>C	G>A	G>A	G>A	G>A	T>C	C>T	
	**Amino Acid Change**								
	23N	M71T	E92K	D119N	V126I	212A	C213R	278N	
H0 ^1^	C	T	G	G	G	G	T	C	AB201628
Ayam Cemani	T	C	A	G/A	G	G/A	T/C	C	This study
H7	T	C	A	G	G	G	T	C	[21]
H8	T	C	A	G	G	A	C	C	[22]
H9	T	C	A	G	G	A	T	C	[18]
H10	C	T	A	G	G	A	C	C	[22]
H11	C	T	A	G	A	A	C	T	[23]
H12	C	T	A	G	A	A	C	C	[20]

^1^ H0—wt allele of Red Jungle Fowl sequence.

## Data Availability

The data that support the findings of this study are available from the corresponding author upon reasonable request. Sequence data are available in GenBank resources (https://www.ncbi.nlm.nih.gov/ accessed on 4 July 2024) under the accession numbers: PP959149 and PP959150.
